# Endoscopic third ventriculostomy in children: problems and surgical outcome: analysis of 34 cases

**DOI:** 10.1186/s41016-020-00228-8

**Published:** 2021-01-06

**Authors:** Md Moshiur Rahman, S. I. M. Khairun Nabi Khan, Robert Ahmed Khan, Rokibul Islam, Mainul Haque Sarker

**Affiliations:** 1 Neurosurgery Department, Holy Family Red Crescent Medical College, Dhaka, Bangladesh; 2 Neurosurgery Department, Bangabandhu Sheikh Mujib Medical University, Dhaka, Bangladesh

**Keywords:** Endoscopic third ventriculostomy, Cerebrospinal fluid, VP Shunt, Communicating Hydrocephalus

## Abstract

**Background:**

Endoscopic third ventriculostomy (ETV) has been established as a viable treatment option for obstructive hydrocephalus of children over 6 weeks of age. ETV in pediatric groups may be unsuccessful due to the failure of absorption of cerebrospinal fluid (CSF) or reclosure of ventriculostomy stoma or due to infection. The exact cause is still debatable. Some issues like failure to eliminate the second membrane during the procedure or formation of the new arachnoid membrane at the stoma are still not clear. This study aims to assess the surgical failure of ETV and its predisposing factors.

**Methods:**

Thirty-four pediatric patients with hydrocephalus were analyzed retrospectively. The patients’ age limit was between 2.5 months and 14 years. This is a retrospective study of 34 patients in a single private hospital between June 2012 and January 2018. Patients having hydrocephalus in pediatric groups more than 6 weeks of age were included in the study.

**Results:**

The mean age of all patients was 51.25 ± 53.90 months and the mean follow-up period was 50.47 ± 20.84 months. Of 34 surgeries, the success rate was 79% and the failure rate was 21%. Within 2 years, the success rate was 68.42% and above 2 years’ success rate was 93.33%. In this series, 7 cases of ETV were re-explored and found ventriculostomy stoma closure in 3 cases, the presence of the second membrane in re-exploration 2 cases, and presence of inflammatory arachnoid membrane in re-exploration 2 cases. The use of dexamethasone around the stoma in inflammatory stoma was useful, having no recurrence. In one patient of the second membrane probably due to absorption failure in communicating hydrocephalus re-exploration was failed and was managed successfully with VP shunt.

**Conclusions:**

Predisposing factors causing ETV failure are ventriculostomy stoma closure by new arachnoid granulation tissues, remnants of the second membrane inside the stoma, CSF absorption failure, infection/high protein in CSF and inappropriate patient selection.

## Background

Endoscopic third ventriculostomy (ETV) has been established for many children with hydrocephalus as an effective treatment [1]. Small and large case series in the medical literature are well documented, but there is still a lack of multicenter data. This lack of data limits our ability to answer important questions regarding ETV complications and efficiency. Precise data on intraoperative events are especially lacking, and the effect these events can have on ETV performance. While the ETV Performance Score (ETVSS) has helped surgeons predict the performance of the operation based on preoperative factors, identifying significant intraoperative factors can further assist decision-making surgeons and perhaps provide insight into the critical technical elements of an optimal ETV [2–5].

The diagnosis of shunt malfunction was made based on clinical symptoms and radiologic signs, including ventricular dilatation on CT. Most CT scans showed only slight differences in ventricular dimensions compared with previous neuroradiologic studies performed when the shunts were functioning, reflecting the loss of brain compliance in these patients after a long-term shunt [6, 7].

## Methods

Data were collected from our database on patients who underwent ETV instead of shunt revision for hydrocephalus in pediatric groups from 2.5 months to 14 years between June 2012 and January 2018. This is a retrospective study of 34 patients in a single private hospital. All patients had undergone recent magnetic resonance imaging (MRI) of the brain for routine ETV in hydrocephalus patients. In emergency cases, a CT scan of the brain was done. Before doing re-exploration for ETV MRI was considered in symptomatic patients. The selection criteria of patients for ETV were based on clinical symptoms and signs and the CT scan of the brain or MRI of the brain in pediatric hydrocephalus group who were more than 6 weeks old.

All statistical analyses were performed using SPSS software version 25. The *p* value < 0.05 was deemed significant.

### Surgical treatment

**Fig. 1:**
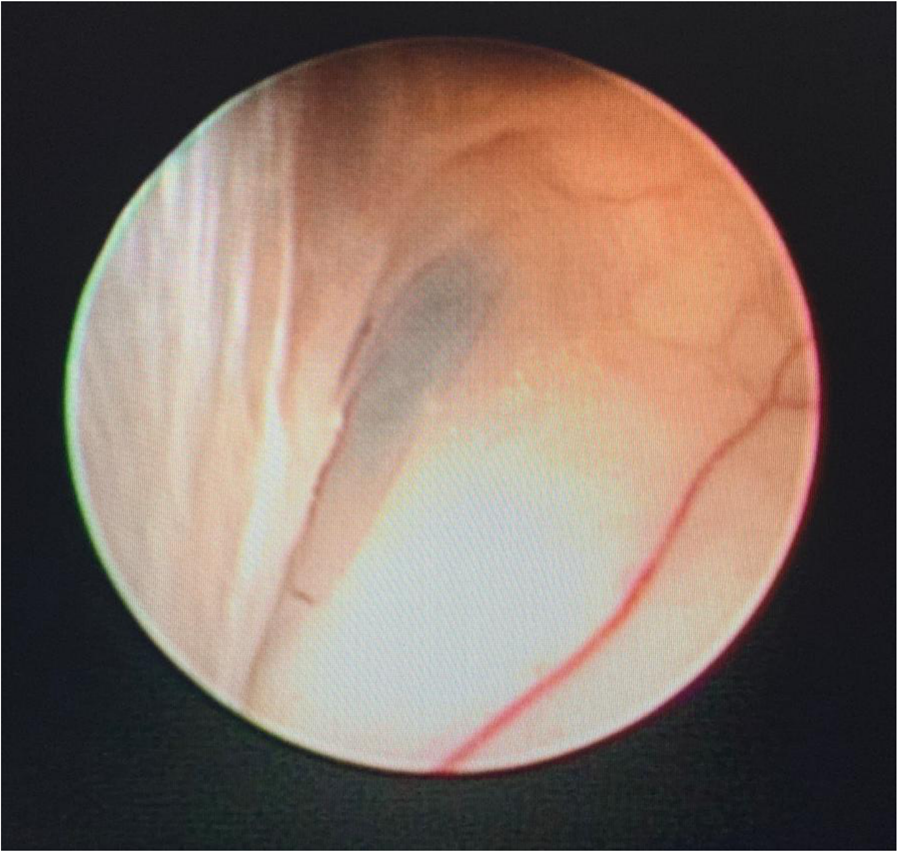
Membranous foramen of monro

One surgeon (first author) used the same technology and equipment for all 34 ETVs. All procedures with patients under general anesthesia and in a supine position have been performed. Head was flexed to avoid air embolism, and the little LOTTA system of KARL STORZ was used for all cases which are autoclave compatible. After a linear incision (2–3 cm) of the skin, an approach was made via a frontal burr hole (1–2 cm in front of the coronal suture and 2.5 cm lateral to the midline) to ensure an adequate trajectory. Foramen of monro was identified and the endoscope was advanced to the third ventricle. In congenital anomaly like membranous foramen of monro (Fig. 1), foraminoplasty and septostomy was adjunct along with the third ventriculostomy. The pre-pontine cistern was inspected and in between infundibular recess and mammillary bodies in the midline a fenestration was made through the bipolar electrode and was enlarged further via Fogarty balloon for sufficient CSF flow. The endoscope was advanced to the fenestrated stoma for inspection of the basilar artery and its branches and also to look for the second membranes. Removal of the previous shunt device was performed during the same surgery. No external ventricular drain was inserted after ETV. For reoperation, the same frontal burr hole was used. In all cases, CSF was sent for culture and sensitivity.

### Postoperative management

Clinical signs and symptoms of intracranial hypertension have been monitored closely in patients. The efficacy of the procedure was determined by the alleviation of clinical symptoms with or without improvements in CT imaging. Measurement of the occipital frontal circumference (OFC) below 2 years of age was recorded by the mother at home weekly and was advised to be noted for follow-up. A postoperative CT scan of the brain was performed at 1 month, 6 months, and 1 year routinely.

Success was defined by the following criteria: (1) no further intervention required to treat hydrocephalus and (2) the absence of signs or symptoms of raised intracranial pressure and (3) decrease in head size [occipito-frontal circumference].

For patients with infection/high CSF, proteins were treated with external ventricular drain (EVD) along with sensitive IV antibiotics according to C/S reports.

**Fig. 2:**
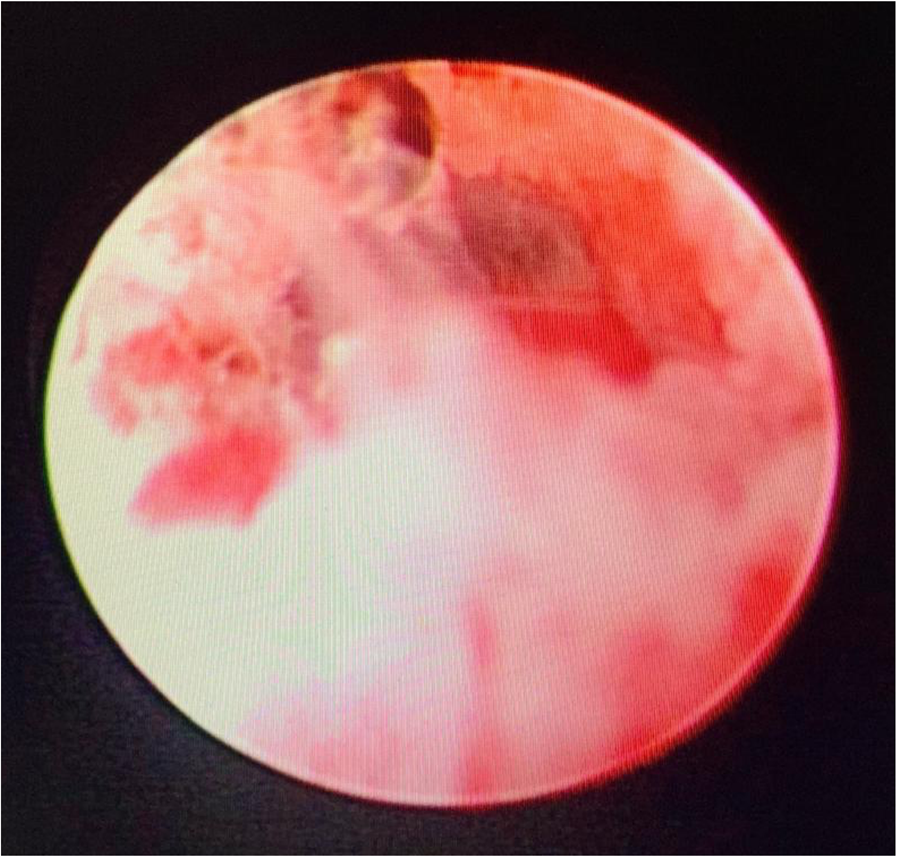
Inflammatory arachnoid membrane/new membrane in ventriculostomy stoma

**Fig. 3:**
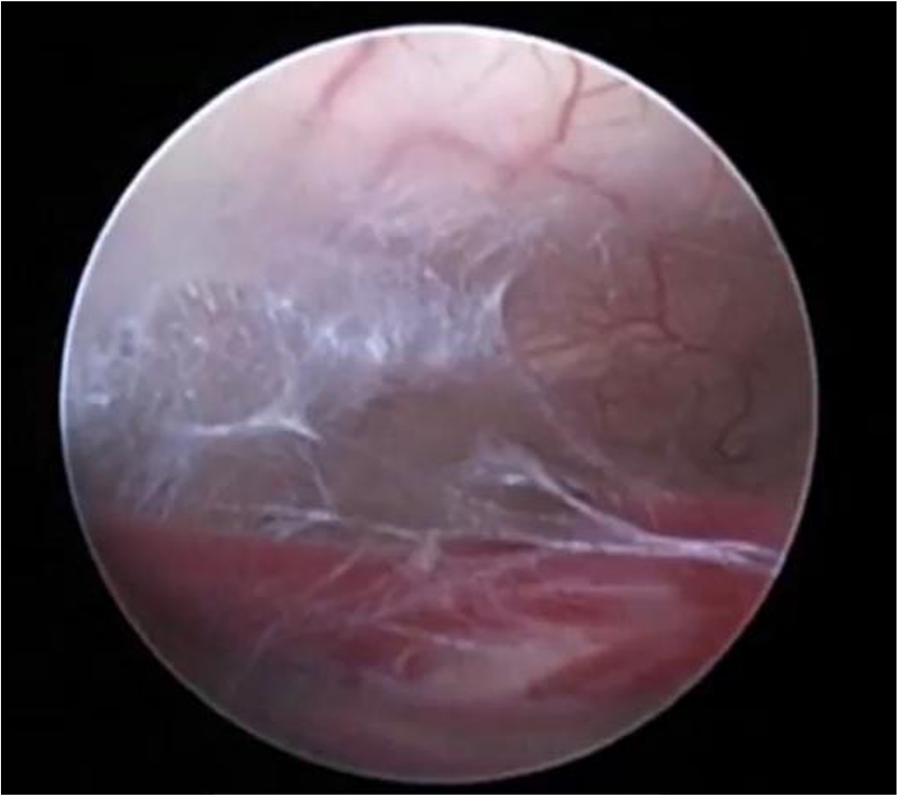
A second membrane inside ventriculostomy stoma

**Fig. 4:**
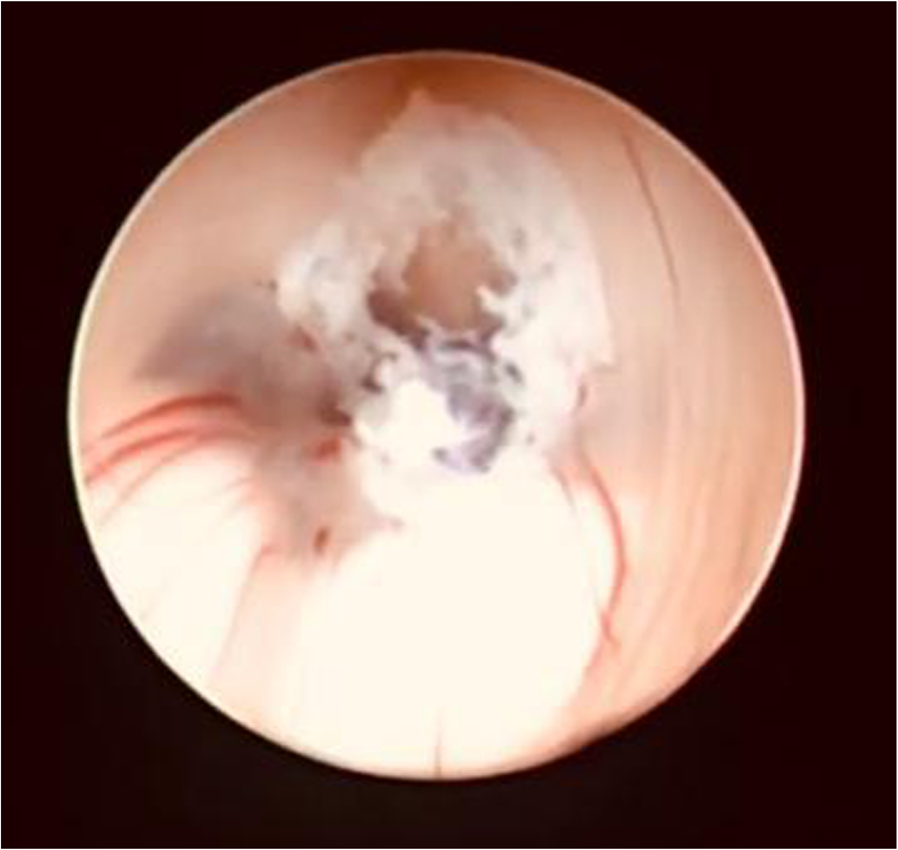
Closure of the fenestration in re-exploration

**Fig. 5:**
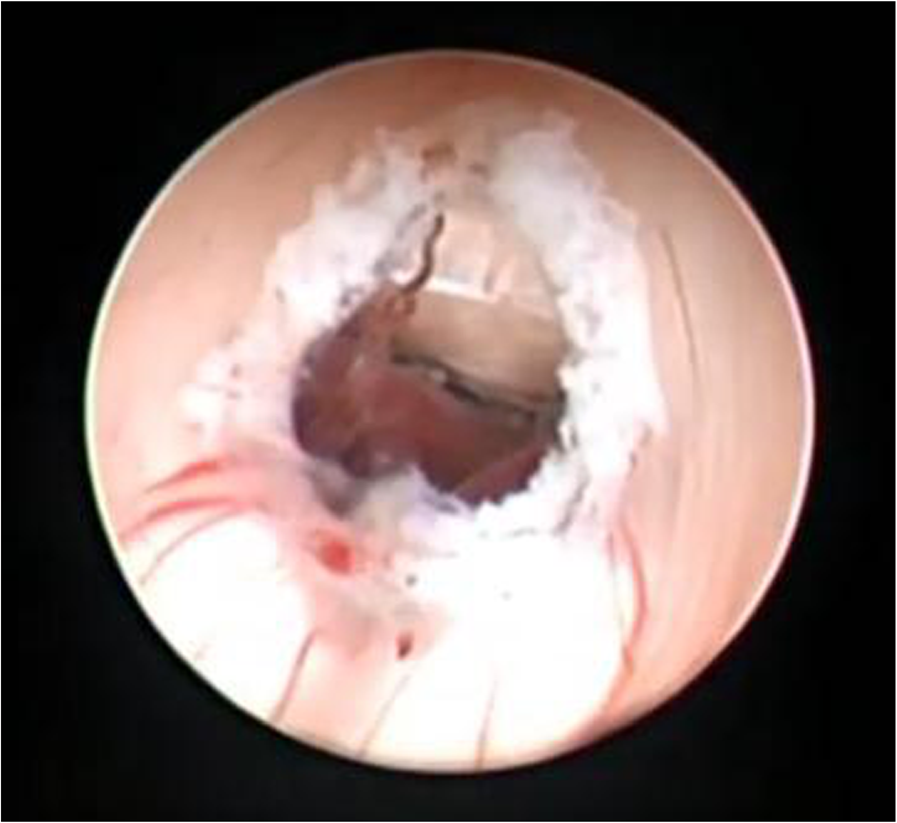
Immediately after balloon dilation of closed stoma

For failed ETV cases, re-exploration and excision of a new membrane (Fig. 2) or second membrane (Fig. 3) with histopathological examination of new arachnoid granulation were carried out and found no pathological tissues. In ventriculostomy closure (Fig. 4), re-exploration and balloon dilatation of closed stoma (Fig. 5) were sufficient to restore the CSF diversion.

In most of the cases, it was sufficient except in one case where the patient had persistent features of raised ICP (intracranial pressure) and was managed with VP shunt in remnants of the second membrane.

### Follow-up

The minimum follow-up period of the patients in the series was 1 year (12 months). They were followed up to a maximum of 7 years (84 months). The mean follow-up period was 50.47 ± 20.84 months (range 12–84 months). Routine postoperative outpatient follow-up appointments were scheduled within 1 week, and then 1 month, 3 months, 6 months, and then every 1 year postoperatively.

## Results

This present study includes a total of 34 patients. Among them, 19 (56%) were male and 15 (44%) were female patients. We included patients between 2.5 months to 14 years in our study. The mean age of all patients was 51.25 ± 53.90 months (range 2.5–168 months). All of them were divided into two age groups: group 1 within 2 years old and group 2 above 2 years old. The mean age of group 1 and group 2 was 10.86 ± 5.95 months and 102.40 ± 42.27 months respectively.

All patients’ clinical profiles are presented in the data chart (Table 1). Etiologically, 5 patients had post meningitic hydrocephalus (HCP) and all 5 (100%) were within 2 years. Twenty patients had congenital hydrocephalus, 9 (45%) were above 2 years, and 11 (55%) were within 2 years. Eight patients had a brain tumor, among them, all 8 (100%) were above 2 years. Two patients had CSF high protein (infection); 1 (50%) was above 2 years and 1 (50%) was within 2 years. VP shunt was in situ in 7 patients before ETV was performed.

**Table 1:** Characteristics of the study population

Variable	***n***(%)	***p*** value
**Sex**		
Male	19 (56%)	0.069
Female	15 (44%)	
**Age**		
2.5 months–2 years	19 (56%)	0.082
2 years–14 years	15 (44%)	
**Diagnosis**		
Post meningitic HCP	5 (14.7%)	0.037
Congenital HCP	20 (58.82%)	
Brain tumor	8 (23.52%)	
CSF high protein (infection)	2 (5.88%)	
VP shunt	7 (20.58%)	
Arachnoid granulation tissue present	2 (5.88%)	
Second membrane present	12 (35.29%)	
**Surgical outcome**		
Successful	27 (79%)	0.013
Unsuccessful	7 (21%)	

### Surgical outcome and prognosis

Surgical procedures were technically successful without intraoperative complications. We performed 34 ETVs (that is, patients who underwent ETV instead of shunt revision for hydrocephalus). Twenty-seven patients recovered successfully because the ETV was functional and effective, and 7 cases were unsuccessful and needed revision surgery. But there was no postoperative mortality in this series. The success rate was 79% and the failure rate was 21%. In group 1, (within 2 years age), the success rate was 68.42% and in group 2 (above 2 years age), success rate was 93.33%. Unsuccessful cases were observed in 6 (31.58%) patients within 2 years and 1 (6.67%) patient above 2 years. In our series, there were no intraoperative complications of endoscopic procedures. However, based on our experience, it is necessary to note that these procedures must be performed only by a surgical team with significant experience in neuroendoscopic operations.

In this series, all patients were followed for at least 1 year between 12 and 84 months follow-up period. During follow-up, the head size, milestone of developments like neck holding, sitting, and walking were evaluated along with psychomotor assessment. Two patients revealed a delay in developmental milestones (walking at 2 years, naming simple objects) out of 7 unsuccessful cases, though the performance in school was normal. A postoperative CT scan of the brain was performed at 1 month, 6 months, and 1 year routinely. Any patient who had increased size of the head, tense anterior fontanelle, or for older children signs of raised intracranial pressure with evidence of increased ventricular size in CT was advised for MRI of the brain. In our study, failed cases were re-explored between 4 weeks and 3 months postoperative period.

The main factors causing failure of ETV are mentioned in Table 2. In this series, a total of 7 cases of ETV were re-explored. The patients with the closure of the stoma and the presence of new arachnoid membranes underwent revision surgery, with good results during the follow-up period. It was found that three cases had ventriculostomy stoma closure and needed revision surgery. Twelve cases were observed with the presence of the second membrane and among them, 2 cases needed revision surgery. The presence of inflammatory arachnoid granulation tissue around the stoma was found in 2 cases and these 2 cases also needed revision surgery. The use of dexamethasone around the stoma in inflammatory stoma was useful in these cases. In one patient of the second membrane probably due to absorption failure in communicating hydrocephalus re-exploration was failed and was managed successfully with VP shunt.

**Table 2:** Predisposing factors causing failure of ETV

Fenestrated prepontine cistern	Total no. of cases	Revision of surgery
Ventriculostomy stoma closure	3	3
Presence of the second membrane	12	2
Presence of granulation tissue around the stoma^a^	2	2

^a^Use of steroid (Dexamethasone 5 mg mixed with normal saline) around the ventriculostomy stoma after balloon dilation in case of inflammatory arachnoid membranes

## Discussion

Obstructive control of hydrocephalus is a challenge and several studies have examined the different treatment methods and their effectiveness [8–10]. ETV is increasingly preferred in the treatment of obstructive hydrocephalus through traditional shunting procedures in selected patients at neurosurgical centers in developing countries with neuroendoscopic expertise. This growing popularity is because ETV provides the patient with the ability to be shunt-free and is successful in treating hydrocephalus regardless of the etiology, patient age, and other factors involved.

In our study, we got a 79% ETV success rate in all patients. Within 2 years (group 1), the success rate was 68.42% and above 2 years (group 2) success rate was 93.33%. Differences in success rate are reported in various literatures [1, 5, 10–12]. According to Heshmati, ETV’s success rate was around 60% which is lower than the recorded rates in pediatric populations [13]. Duru et al. documented their experience with ETV in 51 children under the age of 16; they reported an overall success rate of 80% for all etiologies and ages [14]. Their success rate was 56.2% (9/16) in patients < 6 months of age, 88.9% (16/18) in 6–24 months of age, and 94.1% (16/17) in > 24 months. According to the report of Sodhia et al., the success of ETV in children aged 1–2 years also amounted to 80% [15]. Three patients suffering from aqueductal stenosis, one patient with dandy walker malformation, and one patient with meningitis had excellent outcomes. The adverse result was found in patients with tubercular meningitis who died following surgery the same day. ETV’s goal, and the best objective indicator of a positive outcome to date, is shunt independence. In a study, the univariate and multivariate analysis showed that both hydrocephalus etiology and patient age were relevant factors predicting ETV success [16]. After 2 years of follow-up, Feng et al. found that ETV successfully treated obstructive hydrocephalus in 75% of patients [10]. As reported by Kwiek et al. and Feng et al., the procedure’s success rate typically decreases and is stable at 80% follow-up at 2 years [10, 17].

According to recent studies, the rate of ETV failure with the largest patient populations ranges from 10 to 38.6% [18]. In the present study, seven patients (21%) failed the treatment. But our series did not include mortality. Because of the closing of the ventriculostomy, the involvement of second membrane, and inflammatory tissue, we had a failure rate of 21%. In our study, the failure rate was high in group 1 (patients within 2 years old) than group 2 (patients above 2 years old). One patient suffered an absorption problem. In this order, 7 cases of ETV were re-examined and found; 3 cases of ventriculostomy stoma closure, 2 cases of the second membrane, and 2 cases of the inflammatory arachnoid membrane. For some reports, preceding shunting activity and complex hydrocephalus were stated to be the key causes of ETV failure. So, we agree with Yadav et al. that pre-operative identification of the exact etiology of hydrocephalus can increase the success rate of the ETV and avoid unnecessary surgery [19]. It was strongly suggested in another report that reclosure of the CSF pathway is the factor mainly responsible for ETV failure in obstructive hydrocephalus [20]. Some authors described ETV failure as the shunt placement requirement. According to Kadrian et al., there is a strong effect of patient age on outcome [21]. They recorded extremely low reliability of ETV in infants younger than 1 month. A recent study observed that younger patients with preterm birth had a lower success rate comparing those patients who were mature at the birth time [22]. The correct selection of patients is essential for achieving good results with ETV. Previous shunting operation and complex hydrocephalus were reported to be the main causes of ETV failure.

Predisposing factors that cause ETV failure are ventriculostomy stoma closure by new arachnoid granulation tissues, second membrane relics within the stoma, CSF absorption failure, CSF infection/high protein, and improper selection of patients. The mechanism of failure usually in other studies is the closure of the stoma due to local inflammatory reaction, and its incidence is also related to the underlying pathology. The output of an ETV presents possible risks like CSF leakage, infection, subdural hygromas, and hematomas. ETV has rescued the hydrocephalic patient from shunt dependency and its complications as a foreign body [13]. The complication rate is widely recognized as being linked to the surgeon’s experience. Given the rare but definite risks of the procedure, surgeons must properly select patients with a realistic chance of success. It can also be performed with short postoperative periods in the intensive care unit and with a rather short hospital stay overall, which has a major impact on the well-being of the child. And the diagnosis and treatment of early and late failure of ETV in children also require long-term follow-up.

## Conclusions

The endoscopic third ventriculostomy, in general, is a very effective method of treatment for hydrocephalus in selected pediatric patients. It is linked to a very low rate of permanent morbidity and avoids VP shunt-related morbidity. Predisposing factors causing ETV failure are ventriculostomy stoma closure by new arachnoid granulation tissues and the use of steroids around the inflammatory stoma may be helpful. The presence of remnants of the second membrane also to be addressed and high CSF protein/infection should be corrected and in communicating hydrocephalus ETV may not be useful.

## Data Availability

The datasets used and analyzed in this study are available from the corresponding author on a feasible request.

## Abbreviations

ETV: Endoscopic third ventriculostomy
VP: Ventriculoperitoneal shunt
CSF: Cerebrospinal Fluid
ICP: Intracranial pressure
ETVSS: Endoscopic Third Ventriculostomy Success Score
CT: Computed tomography
MRI: Magnetic resonance imaging
OFC: Occipital frontal circumference
EVD: External ventricular drain
HCP: Hydrocephalus
